# T-helper 17-related cytokines and IgE antibodies during hepatitis A virus infection in children

**DOI:** 10.1590/0074-02760140309

**Published:** 2015-04

**Authors:** Jorge L Trujillo-Ochoa, Karla F Corral-Jara, Griselda Escobedo-Meléndez, Mauricio Realpe, Arturo Panduro, Sonia Roman, Nora A Fierro

**Affiliations:** 1 Unidad de Inmunovirología, Servicio de Biología Molecular en Medicina; 4 Servicio de Biología Molecular en Medicina; 5 Servicio de Infecto-Pediatría, Hospital Civil de Guadalajara Fray Antonio Alcalde, Guadalajara, Jalisco, México; 2 Departamento de Fisiologia; 3 Departamento de Biología Molecular; 7 Departamento de Clínicas Médicas, Centro Universitario de Ciencias de la Salud; 6 Departamento de Medicina Veterinaria, Centro Universitario de Ciencias Biológicas y Agropecuarias, Universidad de Guadalajara, Guadalajara, Jalisco, Mexico

**Keywords:** hepatitis A virus, IgE, Th17, cytokines

## Abstract

We determined the serum IgE levels and T-helper (Th)17-related cytokines during distinct hepatitis A virus (HAV)-induced clinical courses in children. A significantly higher concentration of macrophage inflammatory protein 3α, interleukin (IL)-17E and IL-17F in HAV-infected children with intermediate liver injury compared with those with minor liver damage was found. A reduction in the IgE levels in those patients who showed the highest levels of IL-17F in the group of intermediate liver injury was found. The data suggested that the Th17-related profile is associated with the severity of HAV infection and might play a role on the modulation achieved by HAV during allergies.

Hepatitis A virus (HAV) is an ancient human pathogen and a common cause of enterically transmitted acute viral hepatitis. The virus is highly contagious in both children and adults and highly prevalent in developing countries, where low standards of sanitation promote its transmission (Panduro et al. 2011). A protection against atopy, allergic sensitisation and decreases in IgE levels mediated by HAV infection has been reported. This finding is consistent with the pattern observed in most western countries, where infection by HAV is not common and the incidence of asthma and allergy diseases has increased. Nevertheless, in developing countries where HAV infection remains widespread, allergies are less frequent (Chatenoud & Bach 2011). HAV infection is recognised as the most frequent cause of hepatitis in children (Yeung & Roberts 2010, Escobedo-Meléndez et al. 2012). The infection is acute in nature, but can lead to a broad spectrum of clinical manifestations with varying degrees of hepatic inflammation associated to changes in the concentration of conjugated bilirubin (CB) in the serum and a deregulation in the activity of liver enzymes (Castro-Garcia et al. 2014). CB levels in serum > 2 mg/dL are linked with cholestasis, a condition in which substances normally excreted into the bile are retained (Woolbright & Jaeschke 2012). Thus, it is possible to differentiate cases associated with minor liver damage from those associated with intermediate liver damage during HAV infection (Fierro et al. 2012, Castro-Garcia et al. 2014). Recently, a relationship between severe progression of hepatitis A and protection against allergic diseases has been related with the genetic control of immune receptors (Chatenoud & Bach 2011). However, the exact immune mechanisms responsible for the HAV-mediated protection against the development of allergies remain undefined.

Interleukin (IL)-17 is a cytokine prototype of T-helper (Th)17 cells and is associated with the induction of inflammation (Qu et al. 2013). An increase in the IgE sera levels is negatively correlated with the proportion of Th17 cells in certain allergies, suggesting that Th17 cells may play a role during the development of the allergic process (Hayashida et al. 2011). Th17 cells generate a proinflammatory response and require transforming growth factor (TGF)-β, IL-1β, IL-6, IL-21 and IL-23 to differentiate and secrete cytokines, such as IL-17A, IL-17F, IL-21, IL-22 and tumour necrosis factor (TNF)-α (Kimura & Kishimoto 2010). Recent studies have shown that cytokines related to the subpopulation of Th17 cells mediate the liver damage caused by viral hepatitis, particularly that caused by type B (HBV) and C (HCV) (Lafdil et al. 2010, Wilke et al. 2011). However, at present, the immune mechanisms that are mediated by Th17 cells during HAV infection and their plausible role on the modulation achieved by HAV during allergic processes have not been defined.

## SUBJECTS, MATERIALS AND METHODS

In this study, the concentration of Th17-related cytokines and IgE antibodies in sera samples from 71 acute HAV-infected patients and 40 healthy donors were retrospectively analysed. The blood samples were collected from paediatric patients (< 15 years of age) with acute viral hepatitis diagnosis and admitted to the Fray Antonio Alcalde Civil Hospital of Guadalajara (HCFAA) during 2013 and 2014. Hepatitis was defined as previously described (Fierro et al. 2012). Healthy paediatric donors (< 15 years of age) admitted to the Vaccination Unit of the HCFAA were included in this study. Patients and healthy controls (HC) with liver disease who were being treated with a hepatotoxic drug, participants with chronic hepatitis or autoimmune hepatitis and participants with acute hepatitis E virus, HBV or HCV infections were excluded from the study. The patients and HC had not been vaccinated against HAV. The clinical history and demographic data from participants were collected by a structured questionnaire, as previously reported (Fierro et al. 2012). The serum samples from patients diagnosed with hepatitis were collected between the third-fourth week after the beginning of the infection and were confirmed to exhibit acute infection according to clinical records and by testing for the presence of anti-HAV-IgM and the absence of anti-HAV IgG, abnormal levels of the serum alanine aminotransferase (ALT) and aspartate aminotransferase (AST) (> 38 UI/L and/or > 35 UI/L, respectively) and CB (> 0.3 mg/dL). The ALT, AST and CB levels were measured in the serum samples following routine clinical laboratory procedures. Patients who tested positive for acute HAV infection and who exhibited abnormal values of liver enzymes and CB in the serum were categorised as previously described (Fierro et al. 2012, Castro-Garcia et al. 2014) into the following groups: (i) minor HAV-induced liver injury group, patients who exhibited a CB level of 0.3 to < 2 mg/dL (n = 35), (ii) intermediate HAV-induced liver injury group, patients who exhibited a CB level > 2 mg/dL (n = 36) and (iii) HC, individuals with normal hepatic enzymatic activity and the absence of HAV serological markers (n = 40).

For the evaluation of the cytokines and IgE concentrations in the sera of patients with HAV infection and HC, 25 μL of the sera were tested using a multiplex immunoarray bead assay for IL-1β, IL-2, IL-4, IL-5, IL-6, IL-9, IL-12p70, IL-13, IL-15, IL-17A, IL-17E/IL-25, IL-17F, IL-21, IL-22, IL-23, IL-27, IL-28A, IL-31, IL-33, granulocyte-macrophage colony-stimulating factor (GMCSF), interferon (IFN)-γ, macrophage inflammatory protein 3α [MIP-3α (CCL20)], TNF-α and TNF-β and a separate immunoarray bed for IgE was used (Merck-Millipore, Germany). The immunoarray assays were analysed in a MAGPIX powered by xMAP Luminex Technology with the xPONENT^(r)^ software of EMD (Merck-Millipore). At least 50 events per bead were read for each sample in triplicate wells. The data are presented as the mean ± standard deviation. The nonparametric Kruskal-Wallis test was used to determine the differences between the study groups using GraphPad software v.5.01 (GraphPad Software, USA). p values of less than 0.05 were considered to be significant.

*Ethics* - The blood from the cases and controls was obtained by venipuncture in accordance with approval from the local Ethical Committee of the HCFAA (IRB: HCG/CI-883/09). Informed consent was received from the children's guardians prior to the study. The protocol was conducted in accordance with the Helsinki Declaration of 1975 as revised in 1983.

## RESULTS

A significantly increase in ALT and AST levels was found in HAV-infected children with intermediate liver injury (CB > 2 mg/dL) relative to those with minor liver damage (CB > 0.3 mg/dL - < 2 mg/dL) (Table). We examined the levels of Th17-related cytokines in samples from HAV-infected patients and healthy donors. A low detection rate for these cytokines, including IL-1β, IL-2, IL-4, IL-5, IL-9, IL-12p70, IL-13, IL-15, IL-17A, IL-22, IL-23, IL-27, IL-28A, IL-31, IL-33, GMCSF, IFN-γ and TNF-β, was observed in our study (data not shown). We found a significantly higher concentration of CCL20 (6.821 pg/mL ± 8.397) in HAV-infected children with intermediate liver injury compared with those with minor liver damage (2.592 pg/mL ± 2.938) and healthy donors (0.397 pg/mL ± 0.039) (A in Figure). The Th17-related cytokines, such as IL-17E and IL-17F, were found in significantly higher concentrations in those patients with intermediate liver injury (2.027 pg/mL ± 3.223 and 3.658 pg/mL ± 4.285, respectively) compared with the patients with minor liver injury (0.553 pg/mL ± 0.157 and 0.404 pg/mL ± 0.879, respectively) and healthy donors (0.550 pg/mL ± 0 and 0.297 pg/mL ± 0.681, respectively) (B, C in Figure). No significant differences were found in the IL-21 concentration between the groups (data not shown) and as previously reported (Fierro et al. 2012) the patients with intermediate liver injury exhibited higher concentrations of IL-6 and TNF-β relative to the patients with minor liver injury and the healthy donors (data not shown).

**TABLE:** Demographic and clinical characteristics of patients and controls

Characteristic	Healthy controls	M-HAV-ILI	I-HAV-ILI
n	40	35	36
Mean age (years ± SD)	6.7 ± 2.5	6.5 ± 3.35	8.6 ± 4.2
Gender (% female)	42	57	51
Mean ALT (UI/L ± SD)	20.71 ± 12.14	533.55 ± 529.82	1392.06 ± 1299.90^*a*^
Mean AST (UI/L ± SD)	12.4 ± 7.58	373.44 ± 357.19	1048.72 ± 1189.70^*b*^
Mean CB (mg/d L± SD)	0.145 ± 0.094	1.02 ± 0.57	5.26 ± 2.29
Anti-HAV IgM	-	+	+
Anti-HAV IgG	-	-	-

a: p < 0.001; b: p < 0.05; ALT: alanine aminotransferase; AST: aspartate aminotransferase; CB: conjugated bilirubin; I-HAV-ILI: patients with intermediate hepatitis A virus (HAV)-induced liver injury (CB > 2 mg/dL); M-HAV-ILI: patients with minor HAV-induced liver injury (CB > 0.3 mg/dL - < 2 mg/dL); SD: standard deviation.

**Figure:**
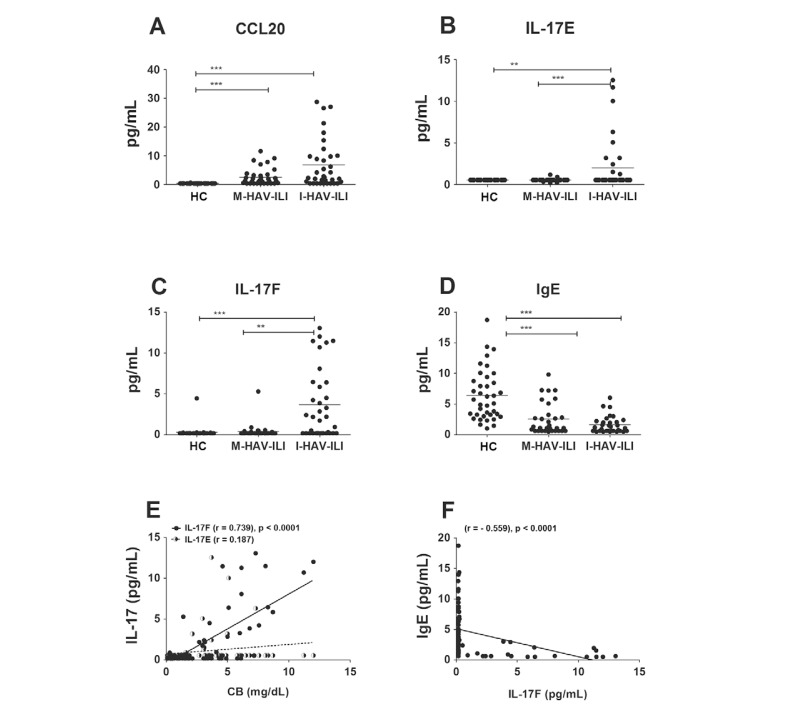
During hepatitis A virus (HAV) infection T-helper 17-related cytokines and IgE are differentially secreted. A: macrophage inflammatory protein 3α [MIP-3α (CCL20)]; B: interleukin (IL)-17E; C: IL-17F; D: IgE sera concentrations determined by multiplex immunoarray assay using xMAP Luminex Technology in sera samples from patients with minor HAV-induced liver injury (M-HAV-ILI) (n = 35), intermediate HAV-induced liver injury (I-HAV-ILI) (n = 36) and healthy controls (HC) (n = 40); E: the Spearman correlation coefficients for IL-17 (IL-17F and IL-17E) and conjugated bilirubin (CB) in M-HAV-ILI, I-HAV-ILI patients and HC; F: IgE and IL-17F in I-HAV-ILI patients and HC were calculated; *: p < 0.05; **: p < 0.001; ***: p < 0.0001. p < 0.05 value was considered statistically significant.

Because a decrease in IgE levels mediated by HAV has been previously reported (Chatenoud & Bach 2011) we reasoned that IgE could be differentially modulated depending on the liver injury induced by HAV. To test this hypothesis, we evaluated the IgE antibodies in the sera from HAV-infected children. We found a significantly lower concentration of IgE in patients with intermediate liver injury (1.619 pg/mL ± 1.390) and patients with minor liver injury (2.553 pg/mL ± 2.59) relative to HC (6.417 pg/mL ± 4.163). No significant differences in IgE values among the groups of minor and intermediated-HAV induced liver injury were found, although data trended towards a reduction in IgE values in patients with intermediate HAV-induced liver damage (D in Figure). We found a profound heterogeneity in the secretion of cytokines, particularly Th17-related cytokines, in the study groups (B, C in Figure). Given that we recently reported that bilirubin plays a role in adjusting STAT function and defining the IL-6, IL-8 and TNF-β profiles during HAV infection (Castro-Garcia et al. 2014), we wondered whether the patients with similar concentrations of IL-17E and IL-17F in the different study groups would have similar values of CB and whether CB can thus play a role in the differential secretion of Th17-related cytokines during HAV infection. Therefore, we analysed the possible correlation between the IL-17E and IL-17F concentrations with that of CB in the serum. No correlation between the IL-17E and the CB concentrations was found. In contrast, the data analysis between CB and IL-17F values revealed a positive correlation, particularly in those patients with CB values > 2 mg/dL (E in Figure). As observed for the cytokines, we found a profound heterogeneity in the IgE levels between HAV-infected patients (D in Figure). We questioned whether Th17-related cytokines, particularly IL-17F could play a role in IgE differential secretion in those patients categorised as intermediate liver injury. To address this hypothesis, we analysed the possible correlation between the IL-17F and IgE levels. The data analysis between IL-17F and IgE values in patients categorised as intermediate liver injury revealed a trend towards a negative correlation (F in Figure).

## DISCUSSION

A polymorphism in the gene that encodes the HAV receptor, *TIM-1 *(157insMTTTVP), is associated with protection from the development of allergies. This polymorphism has recently been related with increased severity of HAV-induced disease (Chatenoud & Bach 2011). Thus, it has been proposed that the low frequency of this polymorphic form of *TIM1 *in the general population may result in the small subset of patients in which infection can lead to severe liver disease (Kim et al. 2011). However, it is unclear how this hypothesis can be reconciled with the observation of a reduced incidence of allergies in regions with a high prevalence of HAV infection, where most of the population presents mild or asymptomatic infection (Chatenoud & Bach 2011). Our findings suggest that it is not only the existence of an anti-HAV response that provides protection, but rather its intensity and quality. Moreover, our data suggest that the Th17-related profile during HAV infection is associated with the severity of disease and may contribute to the development of distinct clinical courses. In particular, in this study, differences in CCL20, IL-17E and IL-17F were found between HAV-infected patients with intermediated liver injury and HAV-infected patients with minor liver injury. This finding is consistent with the proinflammatory profile previously associated with intermediate liver injury, which is characterised by increased levels of IL-6 (Castro-Garcia et al. 2014), a crucial cytokine during the development of the Th17 cellular subpopulation (Kimura & Kishimoto 2010). Interestingly, our data revealed a trend toward a reduction in the IgE levels in those patients who were categorised as intermediate liver injury (CB > 2 mg/dL) and who showed the highest levels of IL-17F, suggesting that during specific HAV-induced clinical courses, increased levels of IL-17F may play a protective role against allergic diseases by reducing the IgE levels. No clinical characteristics of previous or current allergies were found through the retrospective analysis of clinical records of all the patients included in this study (data not shown). Consistent with the potential protective role of Th17 cells against the development of allergies are the negative correlation between IgE and Th17 cells in atopic dermatitis (Hayashida et al. 2011) and the report of an impaired Th17 cell differentiation in autosomal dominant hyper IgE syndrome (Milner et al. 2008). Thus, the possibility to extrapolate to functional role of IL-17 depending on its values during HAV infection in patients with allergies deserves additional investigation. In addition, large-scale studies are necessary to dissect the plausible differences in the incidence of allergies in distinct clinical courses of HAV infection. This approach would enable the definition of the exact role of Th17 cells during the process and the description of potential biomarkers that define resistance or susceptibility to the development of allergies in countries where HAV infection is endemic.
